# The Electrocatalytic Activity of Au Electrodes Changes Significantly in Various Na^+^/K^+^ Supporting Electrolyte Mixtures

**DOI:** 10.1002/smsc.202400042

**Published:** 2024-04-13

**Authors:** Theophilus K. Sarpey, Adrian V. Himmelreich, Kun‐Ting Song, Elena L. Gubanova, Aliaksandr S. Bandarenka

**Affiliations:** ^1^ Physics of Energy Conversion and Storage TUM School of Natural Sciences (Physik‐Department) Technical University of Munich James‐Franck‐Str. 1 85748 Garching Germany; ^2^ Catalysis Research Center TUM Technical University of Munich Ernst‐Otto‐Fischer‐Straße 1 85748 Garching Germany

**Keywords:** double‐layer capacitance, electrolyte influence, laser‐induced current transient, oxygen reduction reaction, potential of zero charge, potential of maximum entropy

## Abstract

The potential of maximum entropy (PME) is an indicator of extreme disorder at the electrode/electrolyte interface and can predict changes in catalytic activity within electrolytes of varying compositions. The laser‐induced current transient technique is employed to evaluate the PME for Au polycrystalline (Au_pc_) electrodes immersed in Ar‐saturated cation electrolyte mixtures containing potassium and sodium ions at pH = 8. Five cation ratios (0.5 M K_2_SO_4_:0.5 M Na_2_SO_4_ = 0:1, 0.25:0.75, 0.5:0.5, 0.75:0.25, and 1:0) are explored, considering earlier studies that unveil cation‐dependent shifts at near‐neutral pH. Moreover, for all electrolyte compositions, electrochemical impedance spectroscopy is utilized to determine the double‐layer capacitance (*C*_DL_), the minimum of which should be close to the potential of zero charge (PZC). By correlating cation molar ratios with the PMEs and PZCs, the impact on the model oxygen reduction reaction (ORR) activity, assessed via the rotating disk electrode method, is analyzed. The results demonstrate a linear relationship between electrolyte cation mixtures and PME, while ORR activity exhibits an exponential trend. This observation validates the PME–activity link hypothesis, underscoring electrolyte components’ pivotal role in tailoring interfacial properties for electrocatalytic systems. These findings introduce a new degree of freedom for designing optimal electrocatalytic systems by adjusting various electrolyte components.

## Graphical abstract

Employing various techniques allows for determining and correlating the potential of maximum entropy (PME), the potential of zero charge, and oxygen reduction reaction (ORR) activity. The impact of electrolyte components (K^+^ and Na^+^ electrolyte mixtures) on Au_pc_ is systematically probed. The results demonstrate a linear relationship between electrolyte cation mixtures and PME, while ORR activity exhibits an exponential trend.

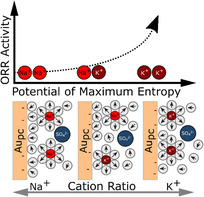

## 1 Introduction

One of the research challenges in electrocatalysis is understanding how the physical or chemical state of the catalyst's surface affects reaction pathways, selectivity, and, ultimately, its impact on the ensuing catalytic performance. Thus, electrocatalysis research primarily focuses on improving the catalytic activity by finding optimized electrode structure and composition conditions.^[^ ^1^, ^2^ ^]^ The performance of a specific electrocatalytic reaction is principally considered to rely starkly on the composition and structure of the electrode surface.^[^ ^3^, ^4^ ^]^ Additionally, an elementary appreciation of the processes occurring at the electrode/electrolyte interface is a prerequisite to successfully developing highly efficient electrocatalytic performance. These are all critical to realizing a cost‐effective, efficient, and sustainable hydrogen economy via electrolyzers, fuel cells, and batteries.

Nowadays, there is a gradual rise of studies focusing on understanding the crucial role electrolyte components, the so‐called “spectator species,” play in optimizing the performance of electrocatalytic systems. Specifically, significant attention has been devoted to investigating the effects of electrolyte pH and alkali metal cations, anions, and even the impact that certain ionic liquids have on the activity of catalytic systems.^[^ ^5^, ^6^, ^7^, ^8^ ^]^ Studies have revealed that cations residing in the electrolyte and, by extension, the electric double layer (EDL), especially in neutral or alkaline solutions, can considerably influence the reaction rate.^[^ ^9^, ^10^, ^11^, ^12^, ^13^ ^]^ For instance, it has been disclosed that the catalytic system activity is closely related to the electrolyte composition and the corresponding local disparate chemical environment.^[^ ^5^ ^]^

Previously, Xue et al. discovered that for electrodes like Pt(111), Pt(221), and polycrystalline Pt (Pt_pc_), the electrochemical hydrogen evolution reaction (HER) activity measured in alkaline electrolytes followed a strict trend of Li^+^ > Na^+^ > K^+^ >Rb^+^ > Cs^+^.^[^ ^14^ ^]^ Notably, the HER current densities of all Pt electrodes in LiOH were fourfold better than those measured in the CsOH electrolyte, regardless of the electrode surface structure. This suggests that alkali metal cations strongly impact the HER activity of different Pt electrodes. Besides the impact of alkali metal cations on the HER, its effect has also been established on other reactions, like the oxygen reduction reaction (ORR), oxygen evolution reaction, and hydrogen oxidation reaction.^[^ ^15^, ^16^, ^17^ ^]^ Strmcnik et al. demonstrated that the noncovalent interactions between hydrated alkali metal cations and adsorbed OH‐species correlate to Pt(111) ORR activities.^[^ ^17^ ^]^ Their work revealed that the ORR activity on Pt(111) follows the trend of Cs^+^ > K^+^ > Na^+^ > Li^+^, which is inversely proportional to the hydration energies of the corresponding cations.

Surprisingly, in particular articles, the authors observed that the electrolyte component effect outperformed the activity effects of different electrode structures and compositions for the same electrolytes.^[^ ^14^, ^18^ ^]^ The electrolyte composition tremendously influences electrocatalytic processes when considering aqueous electrolytes.^[^ ^19^, ^20^ ^]^ In this aspect, the degree of order of the electric double layer is regarded as a critical consideration governing the control of the interface structure and activity and, by extension, the electrocatalytic processes.^[^ ^9^ ^]^ As the interfacial water layer structure becomes more ordered, additional energy is required to rearrange the water dipoles at the interface after the electron transfer.^[^ ^9^ ^]^ The process should be effortlessly accomplished at the potential of maximum entropy (PME), at which the interfacial water molecules have the maximal disorder and, hence, relatively ease the movement of reactant species. Consequently, it is expected that “the closer the PME is to the thermodynamic equilibrium potential of a specific reaction, the faster that reaction should be.”^[^ ^6^, ^21^ ^]^ Stemming from the discussion above, the PME can be defined as the potential where the entropy of the interfacial double layer reaches its peak.^[^ ^18^ ^]^ As a background to understanding the PME concept, it is instructive to highlight Frumkin's earlier work, especially regarding the derivation and further calculation of the potential of zero charge (PZC), which also birthed the PME.^[^ ^22^, ^23^, ^24^ ^]^ Pioneering research work by Lippmann pivotally laid the perfect foundation for Frumkin to build on.^[^ ^25^ ^]^ Since then, several researchers have contributed to further deepening and developing the underlying concepts of the PZC and PME.^[^ ^26^, ^27^, ^28^, ^29^ ^]^

**Figure 1:**
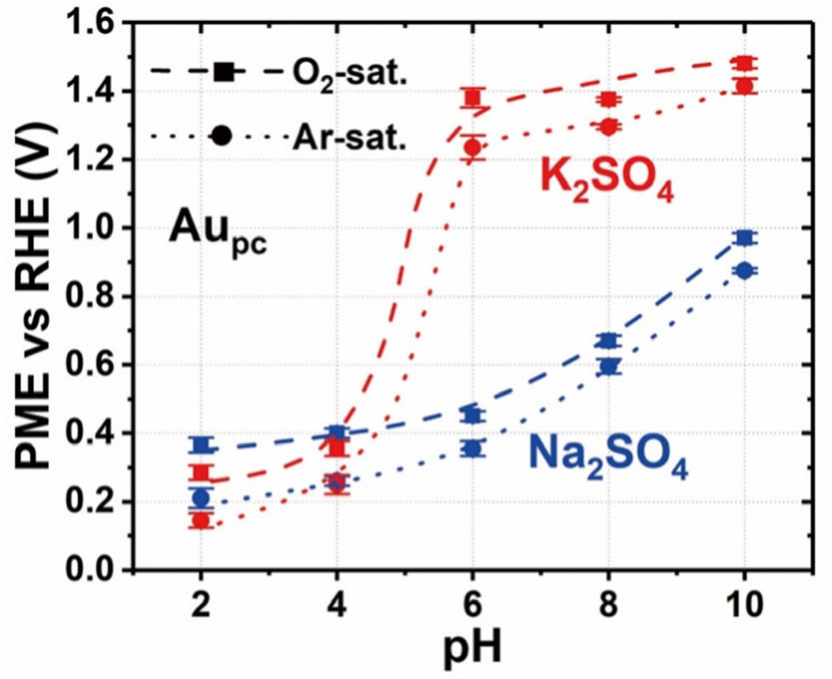
The evaluated PME values for Au_pc_ in Ar‐saturated (dot) and O_2_‐saturated (dash) 0.5 M Na_2_SO_4_ (blue) and K_2_SO_4_ (red) solutions shown as a function of the electrolyte pH. Reproduced with permission from ref. [6] Copyright © (2021), The Authors. Published by Wiley‐VCH GmbH. Open access, published under a CC‐BY license.

Despite recent developments, there is a need for a better understanding of the effect of alkali metal cations on electrocatalytic processes. In this vein, Ding et al. performed a series of laser‐induced current transient (LICT) measurements for the model electrode, gold polycrystalline (Au_pc_) electrodes, to investigate the effect of pH and electrolyte ions (mainly alkali metal cations) on the interfacial processes.^[^ ^6^ ^]^ These were conducted at various pHs (2, 4, 6, 8, and 10) in Ar‐saturated and O_2_‐saturated 0.5 M Na_2_SO_4_ and K_2_SO_4_ electrolytes (**Figure** **1**).

Notably, the PMEs were less dependent on the cation type in the acidic regions. An abrupt modification of the PME is witnessed as the pH is increased from 4 to a near‐neutral electrolyte pH of 6. In particular, the K^+^‐containing electrolyte exhibits a higher sensitivity by rapidly altering its behavior, signaled by ≈1 V increase in PME compared to the Na^+^‐containing electrolyte (cf., Figure 1). The trend was again observed at another near‐neutral pH of 8. Here, the PME measured for the Au_pc_ electrode/Ar‐saturated 0.5 M Na_2_SO_4_ electrolyte was reported to be ≈0.60 V versus reversible hydrogen electrode (RHE). However, using Ar‐saturated 0.5 M K_2_SO_4_ at the same pH yielded a PME value of ≈1.30 V versus RHE (Figure 1).^[^ ^6^ ^]^

This leads to one contemplating whether the sudden PME leap can still be observed for a fixed electrolyte pH of 8 (near neutral condition). At the same time, the cation content is tailored to the molar ratios between sodium and potassium. How does the PME change if electrolyte mixtures of different cation ratios are chosen instead of pure cations? This study is a follow‐up to the findings of Ding et al.^[^ ^6^ ^]^ It aims to resolve some of the puzzling queries from the earlier report. To tackle these questions, finding the PME dependency change between the pure Na^+^‐ and K^+^‐containing electrolytes is essential. This presents a remarkable opportunity to tailor the cation mixture of Na^+^ and K^+^ as electrolytes to obtain an optimal value such that a PME value closer to 1.23 V versus RHE, the thermodynamic equilibrium potential of the ORR, is realized. Hence, an avenue for optimizing the activity via tuning the electrolyte cation concentration can be found. Here, we use the ORR as the model reaction to test this hypothesis.

Moreover, from earlier findings, such a PME, thermodynamic equilibrium potential correlation increases the activity of the electrode/electrolyte processes of the corresponding reaction.^[^ ^30^ ^]^ This empirical law applies to such a mixed electrolyte system. In this respect, it is also essential to measure the ORR activity as a function of the cation molar ratio and elaborate further on the general structure of the interface. This is critical in real‐world applications as the electrolyte choice is also a question of cost‐effectiveness.^[^ ^31^ ^]^ It should be mentioned that Suntivich et al.'s work further affirms the implicit significance of using cation molar ratios, such as Li^+^ and K^+^.^[^ ^13^ ^]^ The authors investigated the ORR activity pattern for a series of KOH and LiOH electrolyte mixtures on Pt/C. They found that increasing K^+^ corresponds to increasing the ORR activity, revealing that the presence of K^+^ could modify the impact of Li^+^ on the ORR activity. The so‐called noncovalent interaction model could not fully explain this finding. Here, using another model electrode, Au, we explore how the alkali metal cation electrolyte mixtures influence its ORR activity.

Even with the remarkable opportunity that cation mixings represent, introducing multiple cation species will immensely complicate the nature of the solid/liquid interface. To explain double‐layer connected features, density functional theory calculations would be insufficient, and instead, ab initio molecular dynamics (AIMD) simulations would be required.^[^ ^32^, ^33^, ^34^ ^]^ Unfortunately, this kind of simulative approach is known for its high computational demand, so no studies describing a mixed cation system have been found so far. In the context of the CO_2_ reduction reaction, Qin et al. performed AIMD simulations to highlight the critical role of spectating potassium cations at gold electrodes in significantly lowering activation energy barriers.^[^ ^35^ ^]^

## 2 Results and Discussion

It is significant to mention that as the hydration energies of the Na^+^ (−365 kJ mol^−1^)^[^ ^36^ ^]^ and K^+^ (−295 kJ mol^−1^)^[^ ^36^ ^]^ cations are much closer to that of the ClO_4_^−^ (−229 kJ mol^−1^) or F^−^ (−465 kJ mol^−1^)^[^ ^36^ ^]^ anions, the SO_4_^2−^ anions with considerably higher hydration energy (−1080 kJ mol^−1^)^[^ ^36^ ^]^ were preferred as they helped to circumvent competition regarding the impact of other species in the electrolyte. In this regard, any variation in the interfacial properties would only be associated with the modifications in the H^+^ concentration and the nature of the alkali metal cations in the investigated systems. Moreover, this study is a follow‐up to the earlier findings, which employed sulfate‐based anions.

### 2.1 Cyclic Voltammetric Measurements

**Figure 2:**
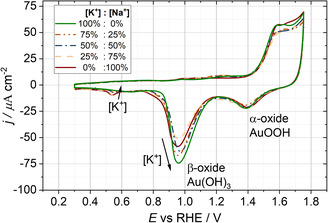
Cyclic voltammetric representations for different mixtures of 0.5 M Na_2_SO_4_ and 0.5 M K_2_SO_4_ electrolytes. This work's near‐neutral pH of 8 shows the alpha and beta oxide phases. Notably, the *β*‐oxide phase increases with increasing values in the potassium molar ratio. However, the third reduction peak is more pronounced with decreasing potassium content.

An overview of the cyclic voltammograms (CVs) recorded before the laser measurements of all ion ratios is illustrated in **Figure** **2**. In contrast to the typical Pt CV in 0.1 M HClO_4_, the definition of a double‐layer region without any faradaic reactions is unclear for Au in the sulfate‐based electrolytes. According to Conway, for *E* > 1.4 V versus RHE, multistep oxidation of the gold surface takes place.^[^ ^37^ ^]^ The steps are a 2D deposition of OH^−^ and O^−^ species on Au, a quasi‐3D surface reconstruction, and the growth of an oxide layer on the Au surface. The structure of the grown oxide layer is pH‐ and anion‐dependent. The two reduction peaks at ≈0.95 and 1.4 V_RHE_ resemble the removal of the oxide layer. Yang and Hetterscheid examined surface oxidation using in situ surface‐enhanced Raman spectroscopy and CV.^[^ ^38^ ^]^ They concluded that mainly two oxide phases, AuOOH (*α*‐oxide) and Au(OH)_3_ (*β*‐oxide), are being formed. The share of these two phases is pH‐dependent, with AuOOH being dominant in acidic solutions, whereas Au(OH)_3_ is formed in alkaline media. It is only in near‐neutral conditions that both phases coexist.^[^ ^38^ ^]^ In previous investigations conducted by Ding et al. variations in the pH of Au in 0.5 M K_2_SO_4_ and Na_2_SO_4_ electrolytes were studied. Similar shifts in reduction peaks were observed and attributed to alterations in proton concentration at the interface.^[^ ^6^ ^]^ Moreover, the measured reduction peak heights for 0.5 M K_2_SO_4_ and Na_2_SO_4_, respectively, were at ≈−40 and −70 μA cm^−2^ for the *β*‐oxide, and −20 and −25 μA cm^−2^ for the *α*‐oxide. The results are comparable to the values obtained in this work (cf., Figure 2).

The similarity between the reference study and the recently obtained CV curves suggests that the electrode/electrolyte system investigated in this work is comparable. Meticulously examining Figure 2 again, one can notice a third reduction peak at ≈0.55 V versus RHE correlating with the decreasing K^+^ concentration. The origin of this peak could be manifold. Generally, the specific adsorption of sulfate could occur in the potential range housing the third reduction peak.^[^ ^39^ ^]^ From the increased oxidation peak areas in the presence of K^+^ compared to Na^+^, one can infer that K^+^ cations promote the oxidation process of Au_pc_. The local protons at the interface exhibit a low concentration for a nearly neutral pH solution. This implies that the local pH at the electrode/electrolyte interface can be easily changed during the oxidation and reduction processes on the Au_pc_ surface. During the reduction process, the protons at the interface can be quickly consumed, which could reduce Au oxide at lower potentials.

As Au_pc_ was used instead of a single‐crystal surface, as many other studies do, grain boundary effects could also play a role. The polycrystallinity also impacts the general CV shape. As nearly all electrode/electrolyte interface‐related parameters, such as reaction rate^[^ ^40^ ^]^ and reaction pathway^[^ ^41^ ^]^ preference, depend on the surface facets, a polycrystalline electrode generates peaks with a larger full width at half maximum.

A cursory view of the CVs recorded in this study also suggests that Na_2_SO_4_ and K_2_SO_4_ cannot be considered equal supporting electrolytes as their individual strengths under the same conditions are vast, agreeing with the findings in the reference study.^[^ ^6^ ^]^ This is further corroborated by the significantly different EDL properties in the presence of these two cations at the same pH of 8. Generally, due to the change of the CV graphs for varying cation concentrations, an effect of the cation mixture on activity measurements seems likely. It is also noteworthy that the pretreatment procedure (see Experimental Section) can influence the CV shape and the surface structure.^[^ ^42^ ^]^ Therefore, the electrode cleaning procedure was repeated before every experiment.

### 2.2 PME Determination for the Cation Mixtures

**Figure 3:**
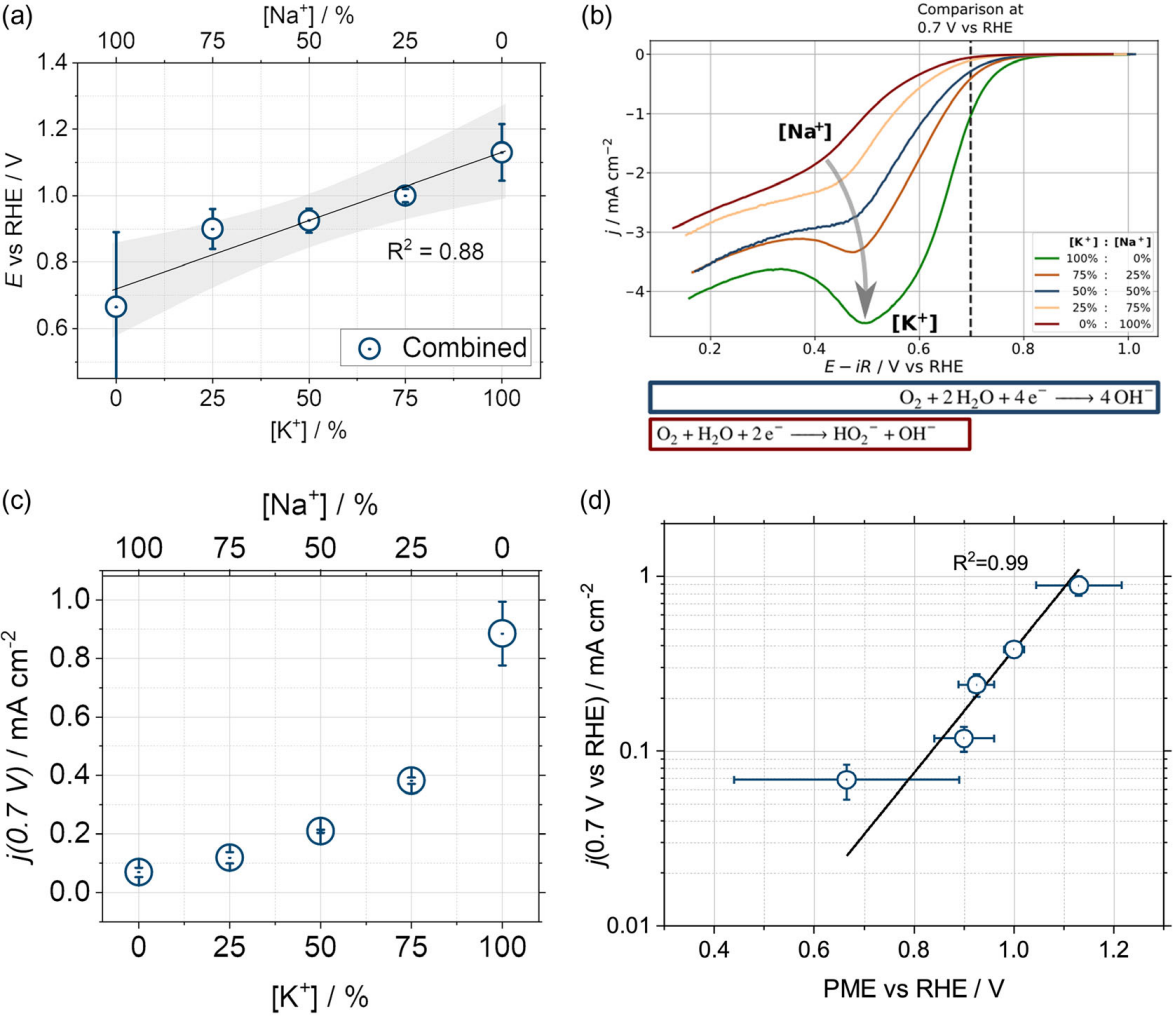
a) Graphical depiction of the combined PMEs versus RHE from this work and the reference study^[^ ^6^ ^]^ plotted as a function of the cation molar ratios. The coefficient of determination is 0.88. b) The polarization curves for the varying cation molar ratios evaluated at 0.7 V_RHE_. c) Specific current densities evaluated at 0.7 V_RHE_ obtained for the cation mixtures in this work. d) Plot of the measured current density at 0.7 V_RHE_ as a function of the PME.

The averaged PMEs between the ref. [6] and the current work for the varying cation ratios are shown in **Figure** **3**a and S6b, Supporting Information. The general influence of electrolyte species (cation molar ratios and their nature) on the PME is fascinating: Just by introducing multiple cations into an electrolyte, the PME, one of the most critical electrolyte‐dependent parameters, appears to be adjustable quasi‐linearly. Such a mixing would be relatively easy to implement in real‐world applications and could be used as a last optimization step in the electrolyte engineering of, e.g., fuel cells or electrolyzers. The implications of an adjusted PME are promising: As the measured PME approaches the ORR thermodynamic equilibrium potential (1.23 V vs RHE) with increasing potassium content, the activity should increase accordingly.

### 2.3 Activity Measurements

Throughout the ORR measurement in O_2_‐saturated electrolytes, the overall measured current decreased for increasing cycle numbers. This behavior is attributed to gold's high surface mobility:^[^ ^43^ ^]^ During the measurement, the surface structure changed accordingly, resulting in a decrease in activity. The gold electrode was, therefore, only cycled thrice per measurement in the oxygen‐purged electrolyte, with the last anodic sweep used as the polarization curve.

Figure 3b presents the *i* *R* and background‐corrected ORR polarization curves with an anodic scan. A Savitzky Golay filter was applied to the data to smoothen noise effects. A clear trend is visible: The ORR rate in 0.5 M Na_2_SO_4_ is the lowest and increases steadily with increasing K_2_SO_4_ content. In addition, with increasing K_2_SO_4_, a local minimum in the polarization curve appears, which can be attributed to a combined effect from the 4e^−^ toward the 2e^−^ pathway and mass transport limitations.^[^ ^44^ ^]^

Lu et al. proved that such a local minimum of the polarization curve is surface facet‐dependent and originates in the adsorption Gibbs free energy change of the reaction intermediates:^[^ ^41^ ^]^ The more pronounced the local minimum in the polarization curve, the higher the tendency toward the peroxide forming 2e^−^ pathway.^[^ ^41^ ^]^ Moreover, the coexistence of two competing reaction pathways complicates examining the double layer's influence on reaction rates. Per the Marcus–Hush–Chidsey (MHC) theory, a curved Tafel plot would be expected if the double layer were responsible for the rate‐determining step.^[^ ^45^ ^]^ However, such a comparison is no longer trivial for multiple pathways. By comparing the ORR currents at a potential of 0.7 V versus RHE, slightly above the peroxide formation potential of 0.682 V versus RHE,^[^ ^46^ ^]^ the PME effect can be analyzed separately from the pathway effect.

### 2.4 Activity–Concentration Correlation

The reduction current at 0.7 V versus RHE plotted as a function of the electrolyte composition is shown in Figure 3c and S6c, Supporting Information. Increasing the K_2_SO_4_ content increases the activity exponentially. The effect is a tenfold improvement for the 0.5 M K_2_SO_4_ electrolyte compared to pure Na_2_SO_4_ and, therefore, quite drastic. Our working hypothesis, *“*The closer the PME is to the thermodynamic equilibrium potential of a certain reaction, the faster this reaction should be*,”* ^[^ ^6^, ^21^, ^30^ ^]^ also applies to this system (Figure 3d). Still, it has to be noted that the acquired activity values are far from being state‐of‐the‐art: The platinum‐based electrodes are known to be better ORR catalysts than Au.^[^ ^47^ ^]^ However, this work aims to investigate the correlation between PME and ORR activity, hence using gold as a model surface with the motivation of extrapolating acquired trends to better‐performing catalysts in future experiments.

Nevertheless, whether the PME alteration is the sole trigger of the change in activity in Figure 3c and S6c, Supporting Information, is still unclear, as various other reasons could also play a role. One probable cause could be a change in the local pH at the interface. Such a pH change would result in a varied potential versus reference electrode (RE). According to the Butler–Volmer equation, the activity would increase exponentially if this hypothetic error on the electrode potential were to increase linearly with the mixing ratio. Additionally, due to the varied cations, the surface coverage with passivating species differs in the case of K^+^ and Na^+^. Besides, as the reaction rate–surface area relation in the Butler–Volmer equation is linear, this cannot explain the exponential increase in activity. Figure 3d demonstrates a plot of the activity at 0.7 V versus RHE against the averaged PME values. As one can see, the activity largely depends on the PME of the system.

### 2.5 Staircase Potentio Electrochemical Impedance Spectroscopy Measurements

The goal was to answer whether the PME for this system corresponds to a more established parameter describing the double layer, i.e., the PZC. The Staircase Potentio Electrochemical Impedance Spectroscopy (SPEIS) technique was employed to determine the double‐layer capacitance, with its minimum (*C*_DL, min_) coinciding with the PZC.

During the analysis of the spectra through the in‐house developed EIS Data Analysis tool,^[^ ^48^ ^]^ it was observed that the linear Kramers Krönig (KK) check resulted in significant errors in the high‐frequency range. Possible wrongdoings in the electrode constellation were investigated and improved. Nonetheless, these improvements did not yield lower KK errors. It is assumed that because of the large surface area of the quartz crystal microbalance (QCM) chip, it is inherently more challenging to apply a uniform AC signal over the entire surface in the high‐frequency regime, hence the increase in KK error.

The electrical equivalent circuit (cf., Figure S6a, Supporting Information) was used to fit the measured spectra. Primarily, a constant phase element (CPE) depicting the double‐layer capacitance was connected in parallel to an *RC* element representing the specific adsorption processes. Subsequently, a resistor denoting the uncompensated resistance was connected in series. The SPEIS data could also define the double‐layer region (i.e., the investigated gray region, spanning 0.6 to 1.1 V_RHE_) as the potential range around the PZC, where the *C*_DL_(*E*) curve is hyperbolic following the Gouy–Chapman theory.^[^ ^49^ ^]^ By contrast, the *n* value of the CPE can be understood as an “ideality” factor or as the “appropriateness” of modeling the double‐layer as a pure capacitor. For *n* = 1, a CPE behaves like an ideal capacitor, whereas a value of *n* = 0 resembles a perfect resistive behavior.^[^ ^50^ ^]^

**Figure 4:**
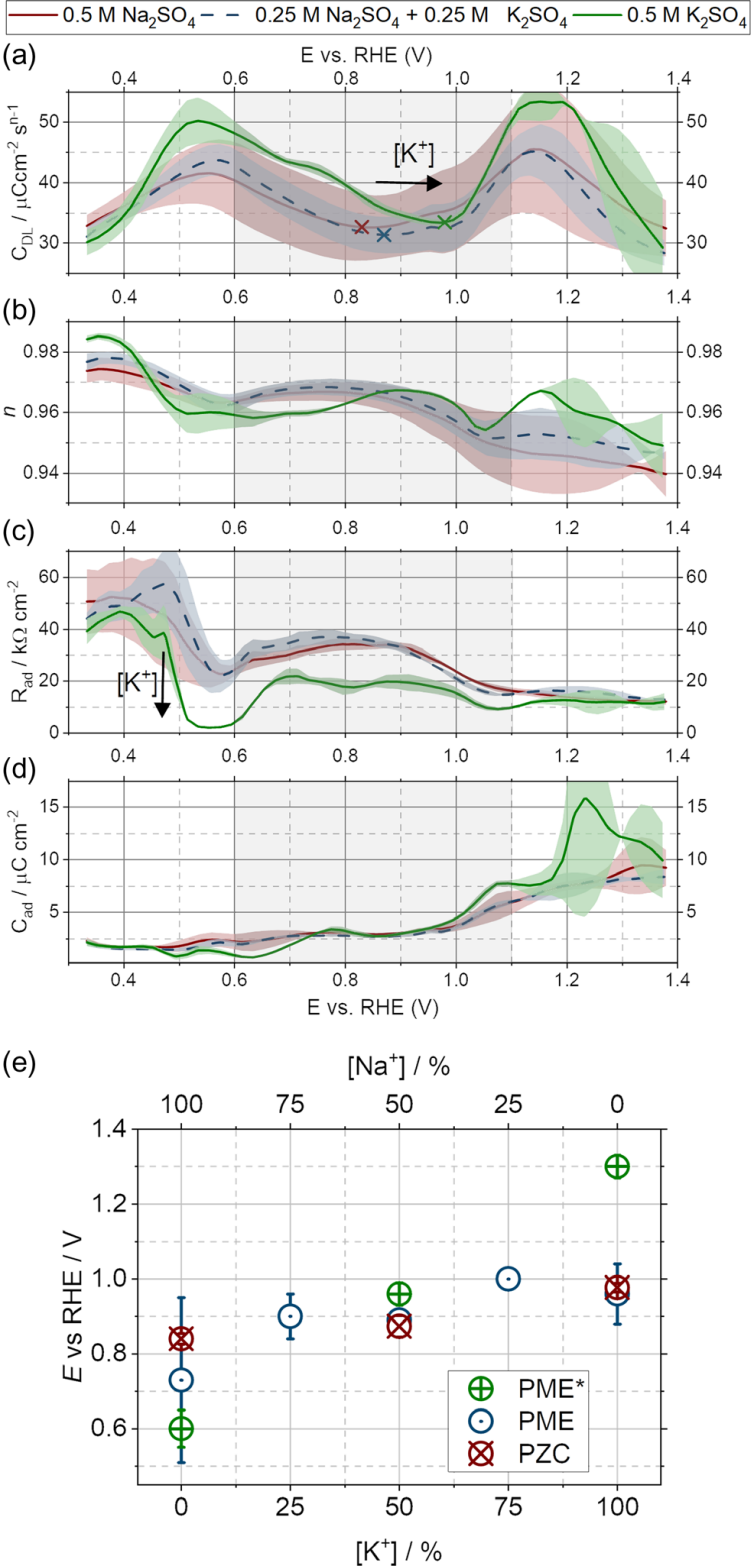
As indicated in the legend, parameters fitted to the SPEIS data of electrolytes averaged over multiple independent measurements. The values in *a*, *c*, and *d* are normalized to the WE area. a) The double‐layer capacitance *C*_DL_. b) The constant‐phase element exponent *n*. c) The specific adsorption resistance $R_{\text{ad}}$. d) The specific adsorption capacitance $C_{\text{ad}}$. The investigated gray area from 0.6 to 1.1 V versus RHE indicates the double‐layer region. e) Depiction and comparison of the separately measured PMEs and PZCs by Ding et al.^[^ ^6^ ^]^ and in this work. The PME from the reference study is marked with*.

Utilizing the linear KK check, data points with an error larger than approximately 4% were excluded from the fitting process. For the fitting, a root mean square fitting error of less than 2% was ensured for all spectra. **Figure** **4**a–d portrays the mean fitted parameters (excluding the uncompensated resistance *R* *_u_*).

By far, the most critical parameter of the equivalent circuit is the double‐layer capacitance in Figure 4a. The curves were “camel‐shaped” for all electrolyte mixtures, while the Gouy–Chapman theory suggests a hyperbolic dependency.^[^ ^49^ ^]^ As deliberated by Shin et al.^[^ ^51^ ^]^ such a shape originates in an interplay of various effects and can be motivated by the following reasoning: For increasing potentials within the anodic bump starting at ≈1 V versus RHE, the anions specifically adsorb at the electrode's surface, leading to a reduced distance *d* of the double‐layer plate capacitor model. Thus, the capacitance increases. Due to the electric field within the inner Helmholtz plane (IHP), the “O‐down” configuration of the water molecules is preferred. For further increasing potentials, the specifically adsorbed anion concentration within the first layer of water molecules increases, increasing the electric field inhomogeneity right at the interface.

This inhomogeneity results in a change from an “O‐down” to an “H‐down” configuration of the water molecules, or, macroscopically speaking, to a saturation of the effective dielectric constant $\varepsilon_{\text{eff}}$ in the inner Helmholtz layer.^[^ ^51^ ^]^ On the other hand, the cathodic bump is caused by a local increase in the dielectric constant. Due to the negative charge on the electrode and the relatively large solvation shell of the cations, the first water layers form a water network within the IHP and outer Helmholtz plane (OHP) that polarizes the interface, leading to an increase in $\varepsilon_{\text{eff}}$. The cation concentration in the OHP increases for more negative potentials, breaking up the water network. Thus, the dielectric constant decreases again.^[^ ^51^ ^]^ It is worth noting that Shin's analysis describes an Ag(111) surface in 3 mM KF instead of a slightly alkaline and more concentrated 0.5 M K_2_SO_4_ electrolyte with a polycrystalline Au surface. Therefore, the reasoning will at least quantitatively vary if applied to the mixed cation system in this work.

As for all electrolytes, the obtained *n*‐values in Figure 4b are ≥ 0.93, the double‐layer behaved almost ideally. The local minima in the *n*‐value versus potential curves have been reported to correlate in some cases with phase transitions of an ionic adlayer.^[^ ^52^ ^]^ Although this adlayer will influence the water dipole orientation, it does not need to coincide with changes in water molecule orientation. Interestingly, for the measured systems, the local minimum *n*‐value at approximately 0.6 V versus RHE coincided with the cathodic capacitance peak of the double layer. The specific adsorption properties of the system *R*_ad_ and *C*_ad_ are presented in Figure 4c,d. It needs to be noted that the adsorption parameters summarize the effects of various potentially adsorbing species, such as SO_4_^2−^ and OH^−^.^[^ ^53^ ^]^

By carefully studying *C*_ad_, one can notice that the adsorption capacitance is almost constant (and also relatively small) until approximately 1.0 V versus RHE, when it starts to increase. This rising capacitance can be understood as the onset potential for forming an oxide layer. Notably, this onset potential is significantly smaller than the value of 1.4 V versus RHE determined through the CV in Figure 2. Nevertheless, as the SPEIS technique is more sensitive toward slight interfacial changes than the CV technique, the value of ≈1.0 V versus RHE is more reliable. In the CV data, the exponential increase in current for *E* > 1.0 V versus RHE hinders the identification of smaller peaks that could also be linked to surface oxidation.

While studying the *R*_ad_ parameter, a local minimum at approximately 0.6 V versus RHE becomes apparent. This resistance minimum was minimal for the pure K_2_SO_4_ electrolyte. It could be correlated with the local maximum, or more precisely, the “almost second zero‐crossing” in the PME curves, which is most pronounced in the case of K_2_SO_4_: The lower the adsorption resistance of a species, the higher its tendency to influence current transients. Similar studies attributed a second PME to the quick adsorption and desorption of protons on Pt electrodes.^[^ ^54^ ^]^ In this work, the investigated system could potentially allow the rapid adsorption and desorption of SO_4_^2−^ instead of protons.

### 2.6 General Electrolyte Trends

In the double‐layer capacitance curve, the PZC corresponds with the local capacitance minimum: If no charge is present on the electrode, the electric field at the solid/liquid interface will be minimal, leading to a minimal polarization of the water molecules. Hence, the capacitance is minimal.^[^ ^51^ ^]^ For many experiments, a PZC slightly more positive than the PME is reported, which is usually explained by some non‐Coulombic interactions of the water dipoles with the *d*‐orbitals of the metal electrode.^[^ ^30^ ^]^ Figure 4e and S6e, Supporting Information, present the PZC values alongside the PME measured in this work and the reference study's earlier work.^[^ ^6^ ^]^

Remarkably, the PME measured in this work aligns with the PZC values within the given standard deviation, contrary to the comparison with Ding et al.'s work.^[^ ^6^ ^]^ Several explanations for this discrepancy are conceivable. First, the PME and PZC may not necessarily coincide; second‐order effects could reasonably place the PZC systematically more positive than the PME. However, this systematic offset does not fully explain the observed data. Besides, if both independent PME measurements were averaged to one dataset (cf., Figure S6e, Supporting Information), most of the values coaligned with the PZCs.

The PZC is the thermodynamic potential at which the solvent restructuring energy is the lowest. In numerous instances, there is a close relation between the PME and PZC, particularly for PME values within the double‐layer region where specific adsorption effects can be entirely disregarded. By contrast, in particular studies, such as the battery‐related research by Scieszka et al.^[^ ^55^ ^]^ or the examination of stepped Pt‐surfaces by García‐Aráez et al.^[^ ^54^ ^]^ more than one PME value has been identified. In those cases, the PME is related to the potential of zero free charge,^[^ ^54^ ^]^ whereas only the potential of zero total charge is accessible utilizing capacitance techniques.^[^ ^30^ ^]^

Finally, comparing the minima of the double‐layer capacitance (*C*_DL, min_) and the PZCs can be fascinating. A comparison of the values for the different mixtures can be found in Figure 4e and S6e, Supporting Information. Figure S6d, Supporting Information, shows a plot of only the PZC values. Due to the variation of the solvation shell, one could expect the capacitance to be the largest in the case of the K^+^‐containing electrolyte and the smallest for Na^+^. However, within the error range for the Na^+^‐containing electrolytes, it is hard to extract any clear trend in the presence of the sulfate ions.

## 3 Conclusion

It was demonstrated that, by systematically varying the sodium–potassium molar ratio, the PME can be linearly tuned, offering great potential for optimizing some electrocatalytic systems. Within the stated experimental conditions, the PME trend aligned perfectly with the empirical PME–activity relation: “The closer the PME is to the thermodynamic equilibrium potential of a certain reaction, the faster this reaction should be.” This correlation could be found for Au_pc_ in Na_2_SO_4_/K_2_SO_4_ for the ORR at pH = 8. The observed difference in activity between the electrolytes based on Na_2_SO_4_ and K_2_SO_4_ is tenfold, a magnificent difference in electrocatalysis. However, the overall measured activities are inferior compared to Pt, which can be explained by the location of gold on the ORR volcano plot.

Most significantly, this study highlights the importance of the right electrolyte choice in electrocatalysis: The MHC theory suggests that solvent reconstruction energy can be rate‐limiting and constrain activities. This reconstruction energy correlates with the water layer stiffness along the interface. Structure‐breaking ions, such as K^+^, can be exploited to minimize restructuring effects. The monotonic change in PME with varying cation ratios demonstrates that aligning the PME with the reaction equilibrium potential can be finely tuned using cation mixtures, surpassing the precision achievable with pure cation electrolytes.

The cation mixing could also be analyzed on an industrial scale to optimize operational and initial capital costs. More information on the Au‐(Na/K)_2_SO_4_ interface could be revealed by interpreting the acquired CV and SPEIS data. By comparing the CVs with the work of Yang et al.^[^ ^38^ ^]^ a difference in the oxide layer formation could be found: The higher the K^+^ content in the electrolyte, the higher the formation share of Au(OH)_3_ instead of AuOOH. No evident trend could be found for the double‐layer capacitance–cation ratio, probably due to relatively high uncertainties of this value for the Na^+^‐containing electrolytes and the seemingly small influence relative to the overall double‐layer capacitance.

Within the SPEIS and LICT measurement uncertainties, the determined PMEs and PZCs coalign, subsequently concurring with earlier published work on their interdependency.^[^ ^30^, ^56^ ^]^ However, the PZC is only an approximation of the PME. Therefore, directly deploying the unique LICT methodology is beneficial for extracting the PME and speculating the PZC's location.

In a nutshell, systematic studies on the impact of the supporting electrolyte mixtures on the interfacial process, i.e., the electrocatalytic performance of Au_pc_ toward the ORR, have been conducted. The presented results and analyses qualitatively and quantitatively elucidate the strong influence of the electrolyte composition on the performance of electrocatalytic systems. Associating the PME with the corresponding changes in the electrocatalytic reactions reflects how the interface structure can control the related electrocatalytic processes. Therefore, determining the PME can be an affordable method to comprehend better the electrochemical processes occurring at the electrode/electrolyte interface.

From an electrocatalytic perspective, the cation mixing approach could be applied to other catalyst/electrolyte systems to prove that the hypothesized extrapolation from a model toward other real‐world systems is valid. In this regard, the cation mixing technique could be employed in the next generation of electrolyzers or fuel cells to optimize these devices further concerning energy conversion efficiency.

## 4 Experimental Section

4.1

4.1.1

##### Cleaning of the Electrochemical Cells

All the experiments reported in this work were carried out using two different electrochemical cells. Before the measurements, all glassware was cleaned with a freshly prepared 3:1 mixture of H_2_SO_4_ (96% Suprapure, Merck, Germany) and H_2_O_2_ (30% Suprapure, Merck, Germany). Then, the glassware was rinsed multiple times with near‐boiling ultrapure water (ρ ≤ 18.2 MΩ cm, Stakpure, Germany). More specifically, the glass cells were initially cleaned once with cold water. Eventually, they were rinsed twice with hot water with a subsequent resting time of ≈5 min to dissolve possible contaminants before the cell was emptied again. Then, the cell was filled again with cold water to cool down the glassware.

##### Working Electrolyte Preparation

The 0.5 M M_2_SO_4_ solutions, where *M* = Na^+^, K^+^, were prepared by dissolving ≥ 99% Na_2_SO_4_ (Sigma–Aldrich) and 99.0% K_2_SO_4_ (EMSURE) in ultrapure water, yielding molar ratios of 0, 0.25, 0.5, 0.75, and 1.0 via the equation, $c=\;\frac{\left[{\text{Na}}_{2}{\text{SO}}_{4}\right]}{\left[{\text{Na}}_{2}{\text{SO}}_{4}\right]+\left[K_{2}{\text{SO}}_{4}\right]}$.

After an appropriate mixing time, a pH meter (Mettler Toledo FiveEasy Plus with a Mettler Toledo LE438 sensing electrode) was immersed into the solution with an equilibration time of ≈5 min to determine the initial (unadjusted) pH value of the solution. 0.1 M solutions of NaOH (≥ 99%, Sigma–Aldrich) and KOH (99.98%, Alfa Aesar) were then added until the pH value was adjusted to 8 without changing the cation ratio.

After the preparation, the measurements were carried out on the same day to minimize the impact of electrolyte decomposition and CO_2_ dissolution. Before the experiment, the setup was saturated with Ar 5.0 (99.999% purity, Air Liquide) for approximately 30 min to minimize the solution's oxygen content. The electrolyte was purged for 30 min with O_2_ 4.7 (Westfalen AG) for the ORR activity measurement.

##### Working Electrode Preparations

Two different working electrodes (WEs) were used in this work. Both offer a polycrystalline gold surface, and their detailed descriptions are provided below. It is vital to note that before each experiment, the electrode and the holder were rinsed multiple times with deionized water to rid the surface of any possible impurities. Figure S1, Supporting Information, highlights all the electrodes deployed in this work.

Following the assembly and preparation of the electrolyte, the cell, and the electrodes on each measurement day, the WE was cycled again to ensure minimal cell hysteresis and a well‐defined surface state. A CV in the potential window between 0.4 and 2.36 V_RHE_ at a scan rate of 50 mV s^−1^ for approximately 50 cycles was performed. Afterward, a second CV in a much narrower potential window was conducted to gain first insights into the gold/electrolyte interface, the stability of the WE, and the experiments’ reproducibility.

##### Au_pc_ QCM Chip

For the LICT and SPEIS measurements, an AT‐cut polycrystalline gold quartz crystal wafer was used. This electrode has a metallic electrode surface area of 1.37 cm^2^ and was installed into a chemically stable holder made from Kynar^[^ ^57^ ^]^ (cf., Figure S1, Supporting Information).

##### Au_pc_ for Activity Measurements

Another WE with a diameter of 5 mm was employed for the activity measurements. Before its application, alumina paste polishing solutions (Micropolish Alumina, Buehler, USA) with granular sizes of 1, 0.3, and 0.05 μm were used to mirror polish the electrode surface. Then, the electrode was cycled in Ar‐saturated 0.1 M HClO_4_ to clean the surface until its CV response congregates electrochemically. It must be added that there is the possibility of screwing or attaching this polycrystalline gold electrode to a rotator. Therefore, the rotating (ring) disc electrode approach was used in this measurement.

##### LICT Measurements: Laser Setup Description

A Quanta‐Ray INDI Pulsed Nd:YAG laser (Spectra‐Physics, USA) generating laser pulses with a width of ≈5 ns, a repetition rate of 10 Hz, a pulse energy of 200 mJ at a wavelength of 532 nm, and a beam diameter of < 10 mm^[^ ^58^ ^]^ was deployed for these set of measurements. The laser's wavelength must be appropriately selected so the laser beam is not significantly absorbed within the electrolyte and glassware. Additionally, the laser wavelength must not excite the electron band structure within the WE and quartz crystal of the QCM holder. In this vein, the employed laser wavelength of 532 nm corresponding to green light is well below the photoelectron emission threshold of gold. A variable motorized beam splitter (VA‐CB‐532‐CONEX, Newport Corporation, USA) partially diverts the beam onto a beam dump to weaken the laser pulses under the electrode damage threshold. The weakened beam is guided through a flat glass window of the working cell and heats the WE. A potentiostat (VSP‐300, Bio‐Logic, France) is deployed to apply potentials and record the current response of the system. The laser, the attenuator, and the potentiostat can be controlled via computer software. A mercury–mercurous sulfate (MMS) electrode was used as the RE, and a flame‐annealed Pt‐wire functioned as the counter electrode. The LICT setup and additional description are provided elsewhere.^[^ ^30^, ^56^ ^]^

##### LICT Measurement Routine

For the PME determination, the LICT technique was employed: A fixed potential was applied to the WE, and after an equilibration time of approximately 15 s, the laser was switched on for ≈4 s, and the current response was recorded. The WE potential was continuously adjusted to get a set of meaningful data. Applying a potential equilibration pause, the measurement of the current response under pulsed laser illumination was repeated until the potential window from 0.33 to 1.33 V versus RHE was screened.

Afterward, a “check‐up CV” in the range of *E* = 0.3 to 1.75 V versus RHE was performed to ensure system and surface stability. The LICT measurement was repeated 2–4 times per measurement day with varying sweep directions: first, a sweep from low to high potentials, then a reversely directed one, and finally, a sweep starting with low potentials again. These steps, such as adjusting the potential and turning the laser on or off, were performed manually. Hence, system equilibration and laser illumination time may vary slightly over an experiment.

##### ORR Activity Measurements

A different electrochemical cell was used for the activity measurements. The WE (PINE Instruments, USA) is mounted on a shaft that can be rotated. As before, an MMS electrode connected through a Luggin capillary was used as the RE. In contrast to the LICT measurements, a gold wire was employed as a counter electrode to exclude Pt deposition on the gold electrode. This was necessitated due to gold's inferior catalytic activity toward the ORR; hence, such a deposition could affect the activity measurements. After the system was assembled, the WE was electrochemically cleaned and pretreated. This procedure was followed by a SPEIS measurement in the potential region of 0.55–0.75 V versus RHE to determine the system's uncompensated resistance. A CV measurement of the background with a slope of 5 mV s^−1^ in the voltage range between *E* = 0.15 and 1.0 V versus RHE was performed. Then, the system was purged for 30 min with oxygen gas (O_2_ 4.7, Westfalen AG). After setting the rotation speed of the rotating disk electrode to 1600 rpm, the actual activity sweep with parameters identical to the background sweep settings was started and run for three cycles. The third cycle was used for further analysis.

##### Impedance Measurements

The SPEIS measurements were performed in the LICT cell with the QCM chip as WE. After the setup preparation and approximately 50 electrochemical cleaning cycles, the electrode was cycled in the window used for the LICT measurements, i.e., from 0.334 to 1.374 V versus RHE, until a steady state was reached. This additional step was introduced to ensure a stable state operation of the system. Afterward, a SPEIS sweep was started at a potential step width Δ*E* of 20 mV and in the same potential window with a frequency range between 1 Hz and 100 kHz. A Pt wire was used and connected in parallel with the RE via a 10 μF capacitor as a dummy RE. For the first two measurements of each electrolyte, the distance of the dummy electrode to the reference capillary was approximately 3–4 cm. For subsequent measurement repetitions of some electrolytes, this nonideal setup was improved by twisting the dummy electrode around the reference's capillary.

## Conflict of Interest

The authors declare no conflict of interest.

## Supporting information

Supplementary Material

## Data Availability

The data that support the findings of this study are available from the corresponding author upon reasonable request.

## References

[1] V. Colic , A. S. Bandarenka , ACS Catal. 2016, 6, 5378.

[2] A. M. Gómez‐Marín , J. M. Feliu , Catal. Today 2015, 244, 172.

[3] W. Li , F. Li , H. Yang , X. Wu , P. Zhang , Y. Shan , L. Sun , Nat. Commun. 2019, 10, 5074. 31699987 10.1038/s41467-019-13052-1 PMC6838099

[4] J. Greeley , I. E. Stephens , A. S. Bondarenko , T. P. Johansson , H. A. Hansen , T. F. Jaramillo , J. Rossmeisl , I. Chorkendorff , J. K. Nørskov , Nat. Chem. 2009, 1, 552. 21378936 10.1038/nchem.367

[5] B. Garlyyev , S. Xue , S. Watzele , D. Scieszka , A. S. Bandarenka , J. Phys. Chem. Lett. 2018, 9, 1927. 29595987 10.1021/acs.jpclett.8b00610

[6] X. Ding , B. Garlyyev , S. A. Watzele , T. K. Sarpey , A. S. Bandarenka , Chem. ‐ Eur. J. 2021, 27, 10016. 34050569 10.1002/chem.202101537 PMC8361723

[7] J. B. Mitchell , M. Shen , L. Twight , S. W. Boettcher , Chem. Catal. 2022, 2, 236.

[8] P. Sebastián , E. Gómez , V. Climent , J. M. Feliu , Electrochim. Acta 2019, 311, 30.

[9] I. Ledezma‐Yanez , W. D. Z. Wallace , P. Sebastián‐Pascual , V. Climent , J. M. Feliu , M. T. M. Koper , Nat. Energy 2017, 2, 17031.

[10] C. M. Gunathunge , V. J. Ovalle , M. M. Waegele , Phys. Chem. Chem. Phys. 2017, 19, 44, 30166. 29105707 10.1039/c7cp06087d

[11] M. M. Waegele , C. M. Gunathunge , J. Li , X. Li , J. Chem. Phys. 2019, 151, 160902. 31675864 10.1063/1.5124878

[12] V. Briega‐Martos , F. J. Sarabia , V. Climent , E. Herrero , J. M. Feliu , ACS Meas. Sci. Au 2021, 1, 48. 36785745 10.1021/acsmeasuresciau.1c00004 PMC9836069

[13] J. Suntivich , E. E. Perry , H. A. Gasteiger , Y. Shao‐Horn , Electrocatalysis 2013, 4, 49.

[14] S. Xue , B. Garlyyev , S. Watzele , Y. Liang , J. Fichtner , M. D. Pohl , A. S. Bandarenka , ChemElectroChem 2018, 5, 2326.

[15] A. Goyal , M. T. M. Koper , Angew. Chem. Int. Ed. 2021, 60, 13452. 10.1002/anie.202102803 PMC8252582 33769646

[16] B. Huang , R. R. Rao , S. You , K. M. Hpone , Y. Song , Y. Wang , W. Ding , L. Giordano , Y. Zhang , T. Wang , S. Muy , Y. Katayama , J. C. Grossman , A. P. Willard , K. Xu , Y. Jiang , Y. Shao‐Horn , JACS Au 2021, 1, 1674. 34723270 10.1021/jacsau.1c00281 PMC8549054

[17] D. Strmcnik , K. Kodama , D. van der Vliet , J. Greeley , V. R. Stamenkovic , N. M. Marković , Nat. Chem. 2009, 1, 466. 21378914 10.1038/nchem.330

[18] A. Ganassin , V. Colic , J. Tymoczko , A. S. Bandarenka , W. Schuhmann , Phys. Chem. Chem. Phys. 2015, 17, 8349. 25412811 10.1039/c4cp04791e

[19] M. C. O. Monteiro , A. Goyal , P. Moerland , M. T. M. Koper , ACS Catal. 2021, 11, 14328. 34888121 10.1021/acscatal.1c04268 PMC8650008

[20] M. Athanasiou , B. Hasa , J. Vakros , L. Sygelloub , A. Katsaounis , J. Chem. Tech. Biotech. 2018, 93, 1542.

[21] X. Ding , D. Scieszka , S. Watzele , S. Xue , B. Garlyyev , R. W. Haid , A. S. Bandarenka , ChemElectroChem 2022, 9, 202101088.

[22] A. Frumkin , A. Gorodetzkaya , Z. Phys. Chem. 1928, 136, 451.

[23] M. Vorsina , A. Frumkin , Dokl. Akad. Nauk SSSR 1939, 24, 918.

[24] A. N. Frumkin , O. A. Petrii , Electrochim. Acta 1975, 20, 347.

[25] G. Lippmann , Ann. Chim. Phys. 1875, 5, 494.

[26] D. C. Grahame , Chem. Rev. 1947, 41, 441. 18895519 10.1021/cr60130a002

[27] V. A. Benderskii , S. D. Babenko , A. G. Krivenko , J. Electroanal. Chem. Interfacial Electrochem. 1978, 86, 223.

[28] J. F. Smalley , C. V. Krishnan , M. Goldman , S. W. Feldberg , I. Ruzic , J. Electroanal. Chem. 1988, 248, 255.

[29] V. Climent , B. A. Coles , R. G. Compton , J. Phys. Chem. B 2002, 106, 5258.

[30] T. K. Sarpey , E. Keleş , E. L. Gubanova , A. S. Bandarenka , in Encyclopedia of Solid Liquid Interfaces (Eds: K. Wandelt , G. Bussetti ), Elsevier, Amsterdam 2024, pp. 43–58.

[31] J. Liu , D. Li , F. Xu , J. Yuan , Z. Ji , Y. Zhao , F. Li , X. Guo , S. Wang , Desalin. Water Treat. 2021, 238, 267.

[32] J. Huang , Y. Zhang , M. Li , A. Groß , J. Phys. Chem. Lett. 2023, 14, 2354. 36848227 10.1021/acs.jpclett.2c03892

[33] R. Haid , X. Ding , T. K. Sarpey , A. S. Bandarenka , B. Garlyyev , Curr. Opin. Electrochem. 2022, 32, 100882.

[34] S. Sakong , A. Groß , Phys. Chem. Chem. Phys. 2020, 22, 10431. 31976502 10.1039/c9cp06584a

[35] X. Qin , T. Vegge , H. A. Hansen , J. Am. Chem. Soc. 2023, 145, 1897. 36630567 10.1021/jacs.2c11643

[36] G. M. Mamardashvili , N. Z. Mamardashvili , O. I. Koifman , Russ. Chem. Rev. 2015, 84, 275.

[37] B. E. Conway , Prog. Surf. Sci. 1995, 49, 331.

[38] S. Yang , D. G. Hetterscheid , ACS Catal. 2020, 10, 12582.

[39] G. J. Edens , X. Gao , M. J. Weaver , J. Electroanal. Chem. 1994, 375, 357.

[40] C.‐Y. Chiu , P.‐J. Chung , K.‐U. Lao , C.‐W. Liao , M. H. Huang , J. Phys. Chem. C 2012, 116, 23757.

[41] F. Lu , Y. Zhang , S. Liu , D. Lu , D. Su , M. Liu , Y. Zhang , P. Liu , J. X. Wang , R. R. Adzic , O. Gang , J. Am. Chem. Soc. 2017, 139, 7310. 28493691 10.1021/jacs.7b01735

[42] R. F. Carvalhal , R. S. Freire , L. T. Kubota , Electroanalysis 2005, 17, 1251.

[43] B. B. Blizanac , C. A. Lucas , M. E. Gallagher , M. Arenz , P. N. Ross , N. M. Marković , J. Phys. Chem. B 2004, 108, 625.

[44] C. Du , Y. Sun , T. Shen , G. Yin , J. Zhang , in Rotating Electrode Methods and Oxygen Reduction Electrocatalysts (Eds: W. Xing , G. Yin , J. Zhang ), Elsevier, Amsterdam 2014, pp. 231–277.

[45] R. A. Marcus , in Nobel Lecture, The Nobel Foundation, Stockholm 1992.

[46] R. Rizo , J. M. Feliu , E. Herrero , J. Catal. 2021, 398, 123.

[47] J. K. Nørskov , J. Rossmeisl , A. Logadottir , L. Lindqvist , J. R. Kitchin , T. Bligaard , H. Jonsson , J. Phys. Chem. B 2004, 108, 17886. 39682080 10.1021/jp047349j

[48] A. S. Bondarenko , Anal. Chim. Acta 2012, 743, 41. 22882822 10.1016/j.aca.2012.06.055

[49] X. Zhao , K. J. Aoki , J. Chen , T. Nishiumi , RSC Adv. 2014, 4, 63171.

[50] M. E. Orazem , B. Tribollet , in Electrochemical Impedance Spectroscopy, John Wiley & Sons, Ltd, Hoboken, New Jersey 2008.

[51] S. J. Shin , D. H. Kim , G. Bae , S. Ringe , H. Choi , H.‐K. Lim , C. H. Choi , H. Kim , Nat Commun. 2022, 13, 57. 35013347 10.1038/s41467-021-27909-x PMC8748683

[52] J. Tymoczko , V. Colic , A. S. Bandarenka , W. Schuhmann , Surf. Sci. 2015, 631, 81.

[53] B. B. Berkes , G. Inzelt , W. Schuhmann , A. S. Bondarenko , J. Phys. Chem. C 2012, 116, 20, 10995.

[54] N. García‐Aráez , V. Climent , J. M. Feliu , Electrochim. Acta 2009, 54, 966.

[55] D. Scieszka , J. Yun , A. S. Bandarenka , ACS Appl. Mater. Interfaces 2017, 9, 20213. 28530796 10.1021/acsami.7b03923

[56] X. Ding , T. K. Sarpey , S. Hou , B. Garlyyev , W. Li , R. A. Fischer , A. S. Bandarenka , ChemElectroChem 2022, 9, e202101175.

[57] Stanford Research Systems , in Operation and Service Manual QCM200 Quartz Crystal Microbalance Digital Controller QCM25 5 MHz Crystal Oscillator, Stanford Research Systems, Sunnyvale, California 2018.

[58] Spectra Physics , in Pulsed Nd and: YAG and Laser User's Manual, Quanta Ray INDI, Mountain View, California 2001.

