# Supplementary material for: The Electrocatalytic Activity of Au Electrodes Changes Significantly in Various Na+/K+ Supporting Electrolyte Mixtures
Source: Small Sci. 2024 Apr 13;4(7):2400042. doi: 10.1002/smsc.202400042 (PMC11934990; doi:10.1002/smsc.202400042)
Supplement: Supplementary file 1 — Supplementary Material [file SMSC-4-2400042-s001.pdf]

Supporting Information

The Electrocatalytic Activity of Au Electrodes Changes Significantly in Various Na^+/K^+ Supporting Electrolyte Mixtures

Theophilus K. Sarpey,+ Adrian V. Himmelreich,+ Kun-Ting Song, Elena L. Gubanova, and Aliaksandr S. Bandarenka**

S1. Analytical Methods for the Potential of Maximum Entropy Determination

S1.1 Peak Extraction

Figure S1 shows some important parts of the cell used in this work**. Figure S2a** shows a typical LICT signal recorded per voltage step for an arbitrary electrolyte (here 0.5 M K_2SO_4). The current is measured after setting the potential to a fixed value, in this case, a relatively high potential of *ca.* 1.07 V *vs.* RHE. The exponentially decaying current during the first seconds constitutes the double-layer charging of the working electrode (WE) as the current approaches a steady state. Now, the pulsed laser is being switched on, and the typical current transients in the form of sharp peaks are clearly visible. Since a positive peak can be understood as a positive current entering the electrode during cooling to reorder the double layer, the electrode charge is positive for the voltage presented, i.e.,1.07 V_{RHE}. By magnifying the laser pulse region, it becomes apparent that the current transients are not constant over time. They sometimes change from negative to positive within one pulsing.

Additionally, the pulse height largely varies. There could be diverse reasons behind this occurrence. Generally, one could divide the reasons into the measurement setup and those based on the physically meaningful behavior.

On the system side, a varying pulse intensity of the laser over the pulse train or a sluggish potentiostat response could explain peak variations. Pulse energy variations were determined to be unlikely.[1] The settings were slightly varied to investigate the potentiostat's influence. Two settings, namely the chosen bandwidth and the current range settings, impact the recorded signal quality (details shown elsewhere). [2] However, they do not affect the general variation of the peak height over time. It is assumed that this variation is likely due to the heating of the electrode. Therefore, to at least ensure the same surface heating for every potential, the pulsing duration must be kept as constant as possible, and not all but only a small number of

pulses in a row are used for further analysis. **Figures S2b** and **c** show that 5 pulses were extracted as a compromise between pulse height variation and statistical fluctuations.

S1.2 Background Correction

After cropping out five peaks, a background current removal must be applied. Ideally, this background current should be zero if no concurrent electrochemical reaction occurs. Due to traces of oxygen, some small double-layer charging currents, and other minor side reactions taking place, there could be a small background. The median of the current time series is computed to approximate the background current.

S1.3 Averaged Transients and Possible Descriptors

Exemplary current transients for various potentials plotted for the 0.5 M K_2SO_4 electrolyte are presented in **Figure S3**. For low potentials from 0.35 V to 0.88 V *vs*. RHE, the transients are clearly negative, although, at 0.88 V_{RHE}, the transient seems slightly bipolar. At 1.05 V *vs*. RHE, the transients are positive, and the peak size and the bipolarity appear to increase with further increasing potentials. Here, the creation of an adlayer, as described in the CV results, hampers the usefulness of the LICT method and influences the transients' shape. Therefore, by carefully studying Figure S3, the PME can be determined to be located between 0.88 V and 1.05 V *vs*. RHE.

More potentials need to be included and evaluated for a more precise determination of the PME. To implement this efficiently, a descriptor must be defined to map the current transients to a single value. Notably, negative descriptor values indicate negative transients, while positive values correspond to positive transients. In the past, two different descriptors were used for pulse evaluations.

S1.3.1 Extreme Current

This method was used for most of the earlier published papers.[3,4,5,6] The descriptor is identified as the current value, where the absolute value of the current reaches its extremum.

S1.3.2 Integrated current

This method resembles the attempt to find a physically meaningful descriptor and was used in a study published by Auer *et al*.[7] It is defined as the time integral of the current over the range of the cropped pulses. Thus, it can be interpreted as the amount of free charge on the electrode:

Due to the laser heating, the polarized inner Helmholtz layer loses its order, i.e., the dielectric constant of the equivalent plate capacitor decreases. This leads to a decrease in the double-layer capacitance. Hence, according to the definition of $Q = CU$, in a potentiostatic environment, the free charge on the electrode will follow the change in capacitance.

Due to the automated analysis procedure, both methods could be applied to the measured transients.

S1.4 Measurement Results

The PME per sweep is given by the negative to positive zero-crossing of the sweep. To compare the two descriptors' effect on the PME determination, the above-discussed sweep of 0.5 M K_2SO_4 is shown separately in **Figure S4**. In this case, the resulting curve shapes of both descriptors are comparable. However, when all sweeps are examined, this may not always hold, making it challenging to make general statements about the curve shape's similarity. The precise curve shape also appears to depend on the peak selection. Overall, most curves are slightly s-shaped. The comparison of the extracted PME values yields a rather unsatisfactory result: The PMEs determined with the integration method are consistently more negative than those where the extreme current method was used. However, this result is not entirely unexpected: If one assumes that two competing processes happen simultaneously at the electrode on fast time scales with opposite currents, the current response will be given by the sum of these two processes. According to Auer *et al.*[7] and Benderskii and Velichko,[8] electrochemical cells with relatively high internal resistance will slow down the double-layer relaxation, resulting in an exponential current transient shape per process:

$$j(t) = \begin{cases} 0, & for\ t < 0 \\ A_1 e^{-t/\tau_1} + A_2 e^{-t/\tau_2}, & for\ t \geq 0 \end{cases} \qquad \text{(Equation 1)}$$

Examples of a second process occurring with the water dipole restructuring could be the quick desorption of an anion as OH^-[9] or SO_4^{2-} and diffusion layer restructuring. Another factor that can influence the system is the thermodiffusion potential, which results from a potential drop caused by temperature gradients within the bulk electrolyte.[8] Notably, this effect becomes more pronounced at higher ionic concentrations.

As depicted in **Figure S5**, where the pulse parameters of Equation 1 were chosen by eye to arrive at a shape like in experiments, this can sometimes lead to contradicting transient classifications. For bipolar pulses, the potential, where the integrative descriptor yields q = 0 C

cm^{-2}, can be understood as the state where the relaxation of one process exactly counterbalances the other.

Hence, as Auer *et al.*'s[7] work revealed the integration method can only be used for non-bipolar transients yielding a linear q vs. E curve. This is only the case for the measured data for a narrow potential range around the zero-crossing. Especially for the electrolytes with a higher K_2SO_4 content, there seems to be, apart from the zero-crossing, almost a "zero-touching" at approx. 0.5 V *vs*. RHE. This *local maximum* of the transient curves seems to correlate with a slight reduction bump in the CV (Figure 2) and a decrease in the specific adsorption resistance.

However, in the CV, the most prominent peak is given by the pure Na_2SO_4 solution, and in the case of the LICT, the local maximum is most pronounced for K_2SO_4. Therefore, the CV-LICT data correlation could be purely coincidental. By contrast, the extreme current descriptor is less influenced by slower secondary processes and appears more robust to circumvent transient classification as positive or negative. Also, errors in the background correction have a less significant effect on the extreme current descriptor. Lastly, this work is a follow-up project to reference,[5] where the extreme current method was used. Therefore, this work employed the extreme current descriptor for further analysis and discussion due to the stated advantages. Nonetheless, the extreme current descriptor is not connected to a direct physical quantity, as is the case for the integrative descriptor, restricting the analysis of the LICT data to the position of the PME.

S2. Supporting Figures

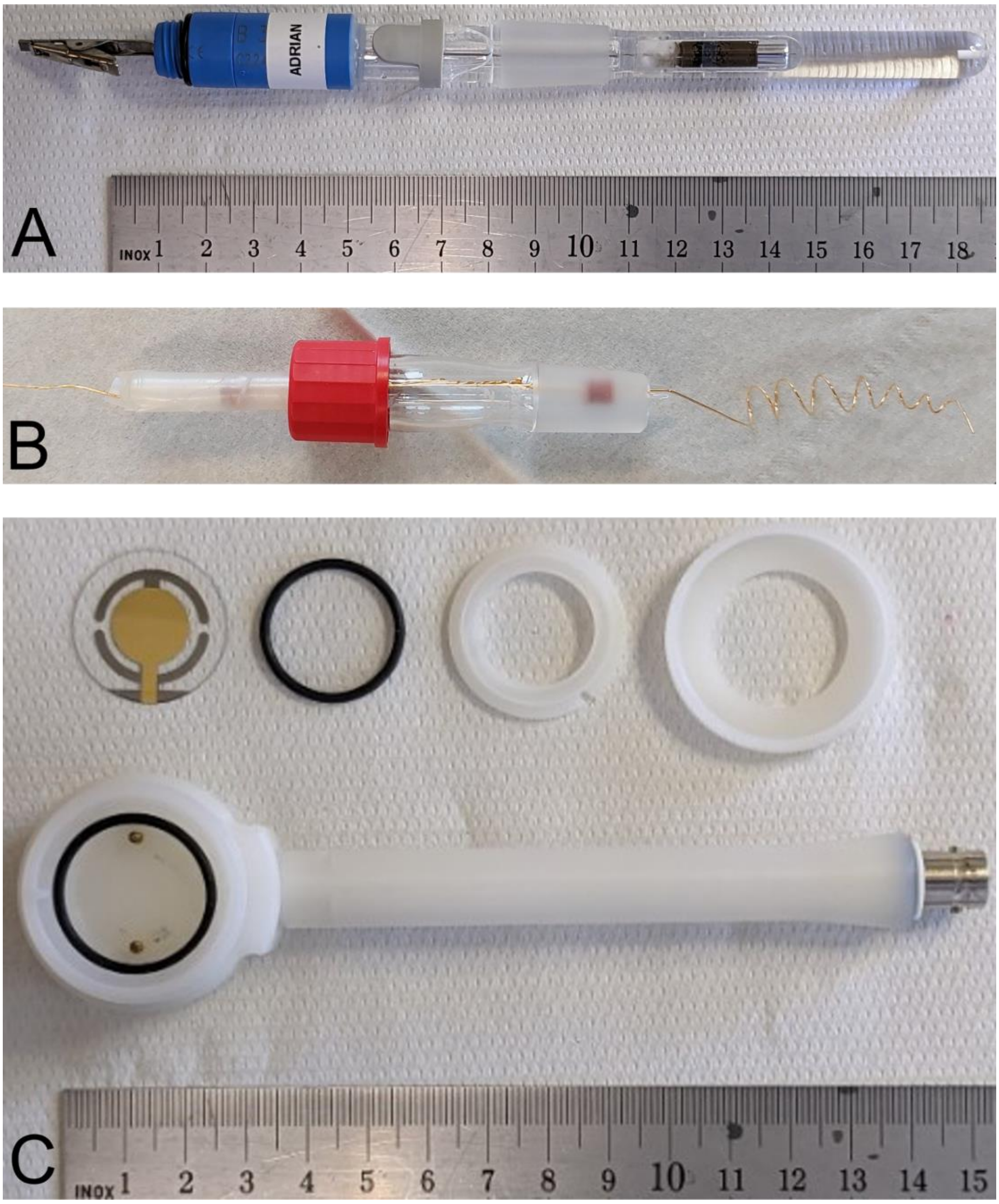

Figure S1. a) MMS electrode that functioned as a reference electrode in all the experiments. b) Au CE used for the activity and the SPEIS measurements. c) Unassembled QCM chip electrode with its holder and rings used as a WE in the LICT and SPEIS experiment.

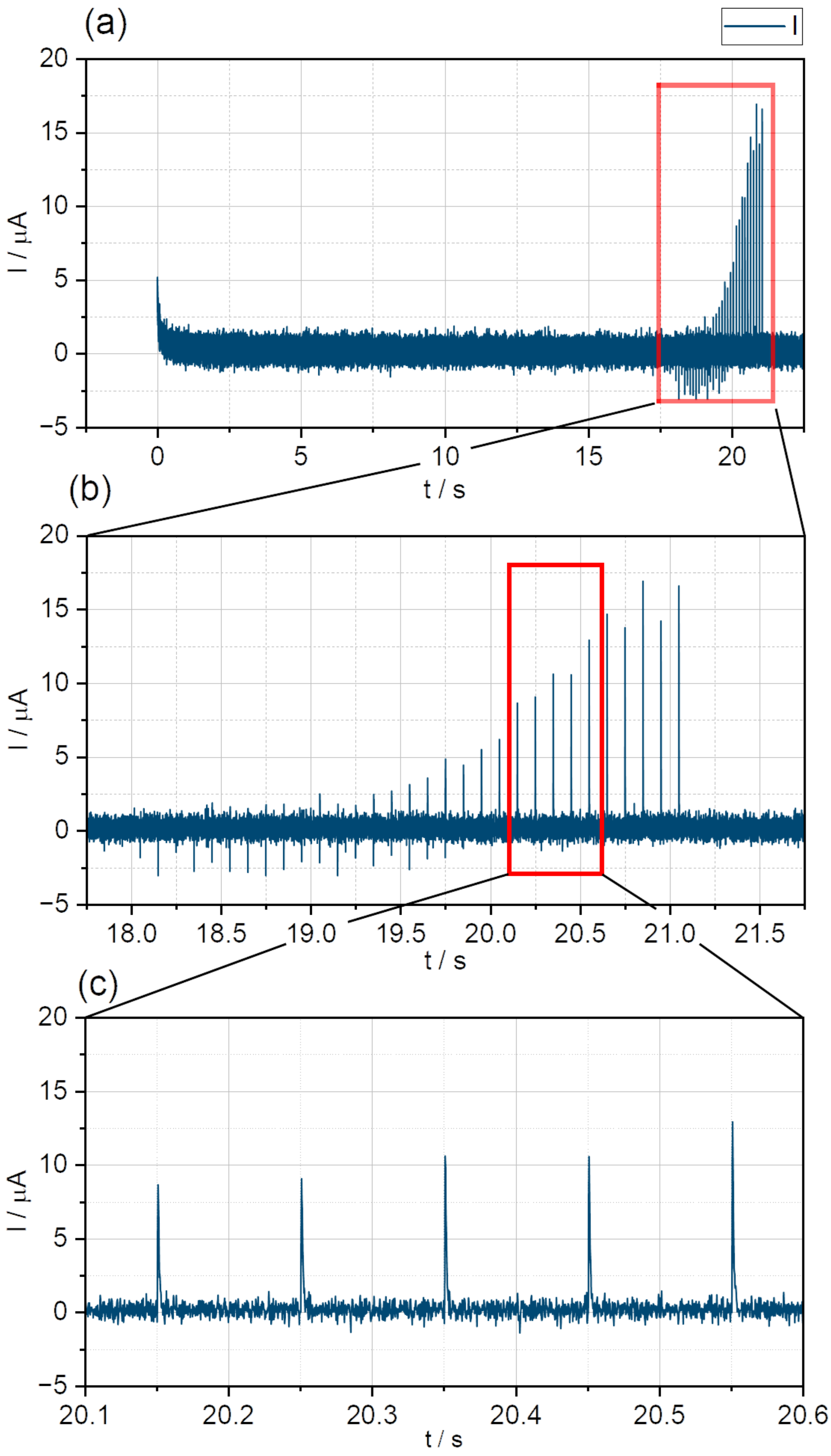

Figure S2. a) Example of an arbitrary data file recorded at a relatively high overpotential of *ca*. 1.07 V *vs*. RHE. for 0.5 M K_2SO_4. Slight deviations in the current values of b) and c) are purely due to displaying precision of origin.

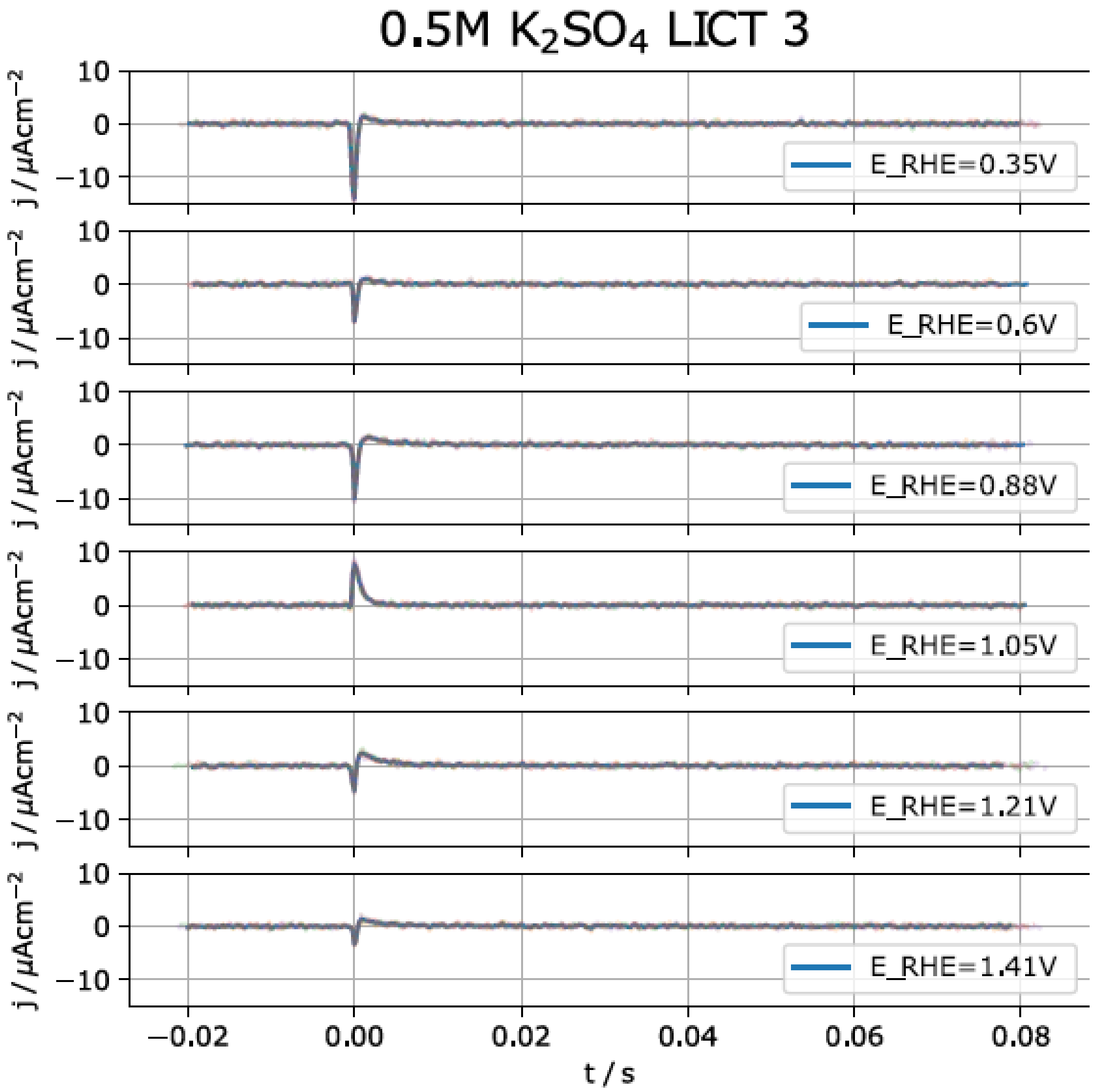

Figure S3. Averaged LICT pulses for varying WE potential. The current transients change their sign between 0.88 V and 1.05 V *vs*. RHE. An arbitrary sweep in 0.5 M K_2SO_4 is displayed as an example.

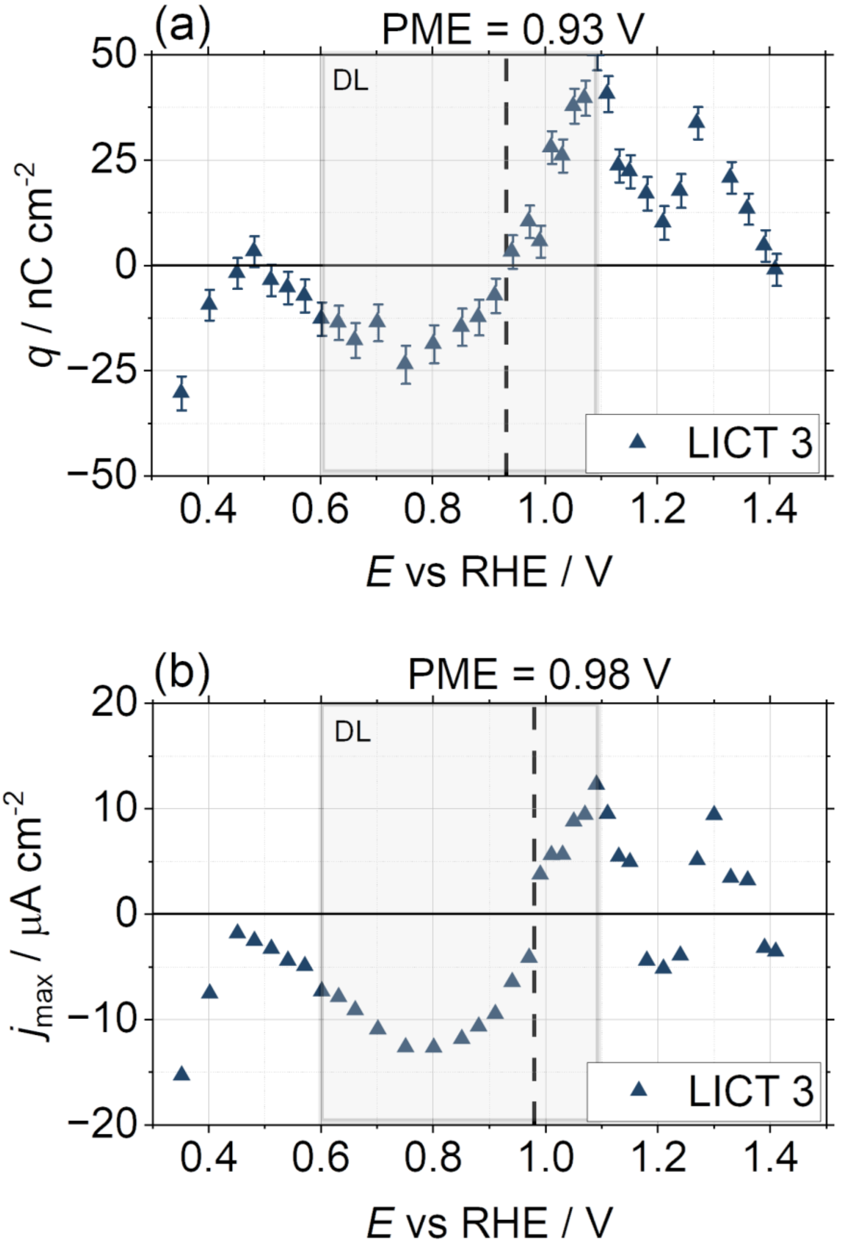

Figure S4. Resulting descriptor values for the exemplary sweep. a) Integrative descriptor. b) Extreme current descriptor. The double-layer region, determined through SPEIS, is highlighted in grey. Depending on the descriptor choice, slightly different PME values were obtained.

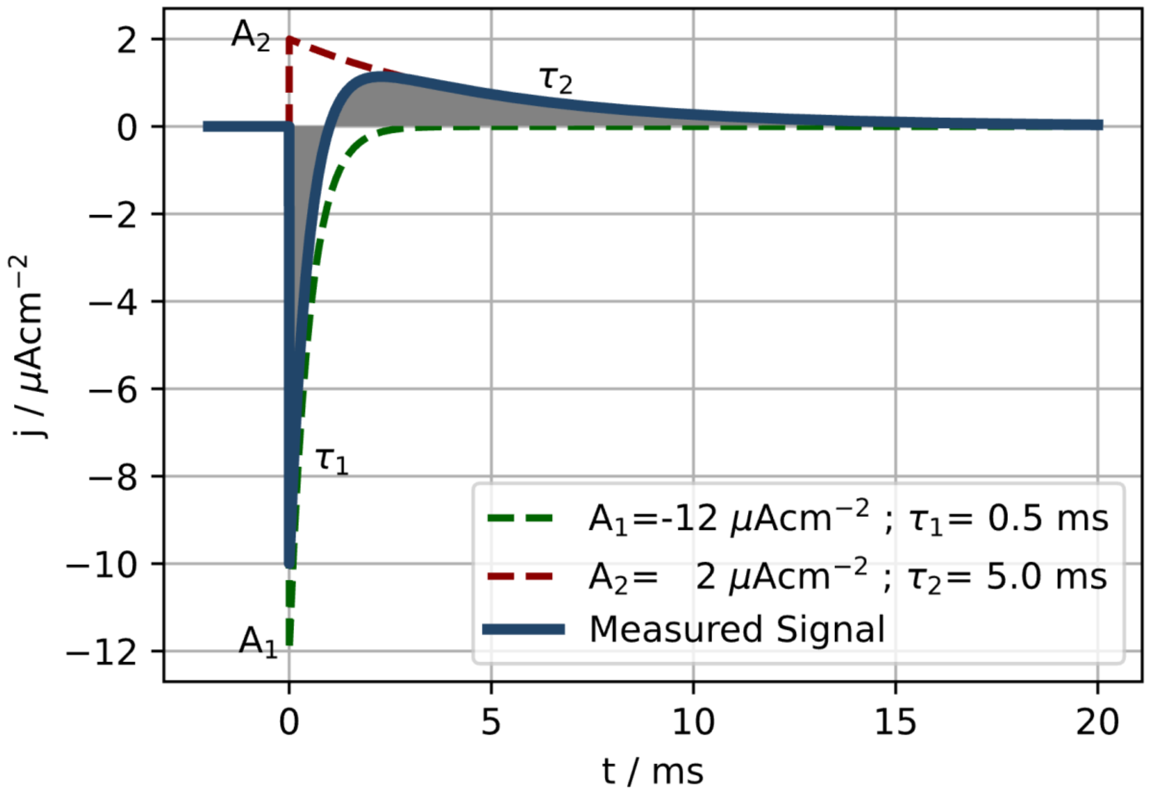

Figure S5. For bipolar peak shapes, the two descriptors yield different peak classifications. In this "exaggerated" example, two processes with different peak heights and relaxation times overlap. The extremum descriptor yields a negative transient of -10 µA cm^{-2} instead of -12 µA cm^{-2}, whereas the integrative descriptor yields a positive transient of 4 nC cm^{-2} instead of -6 nC cm^{-2}.

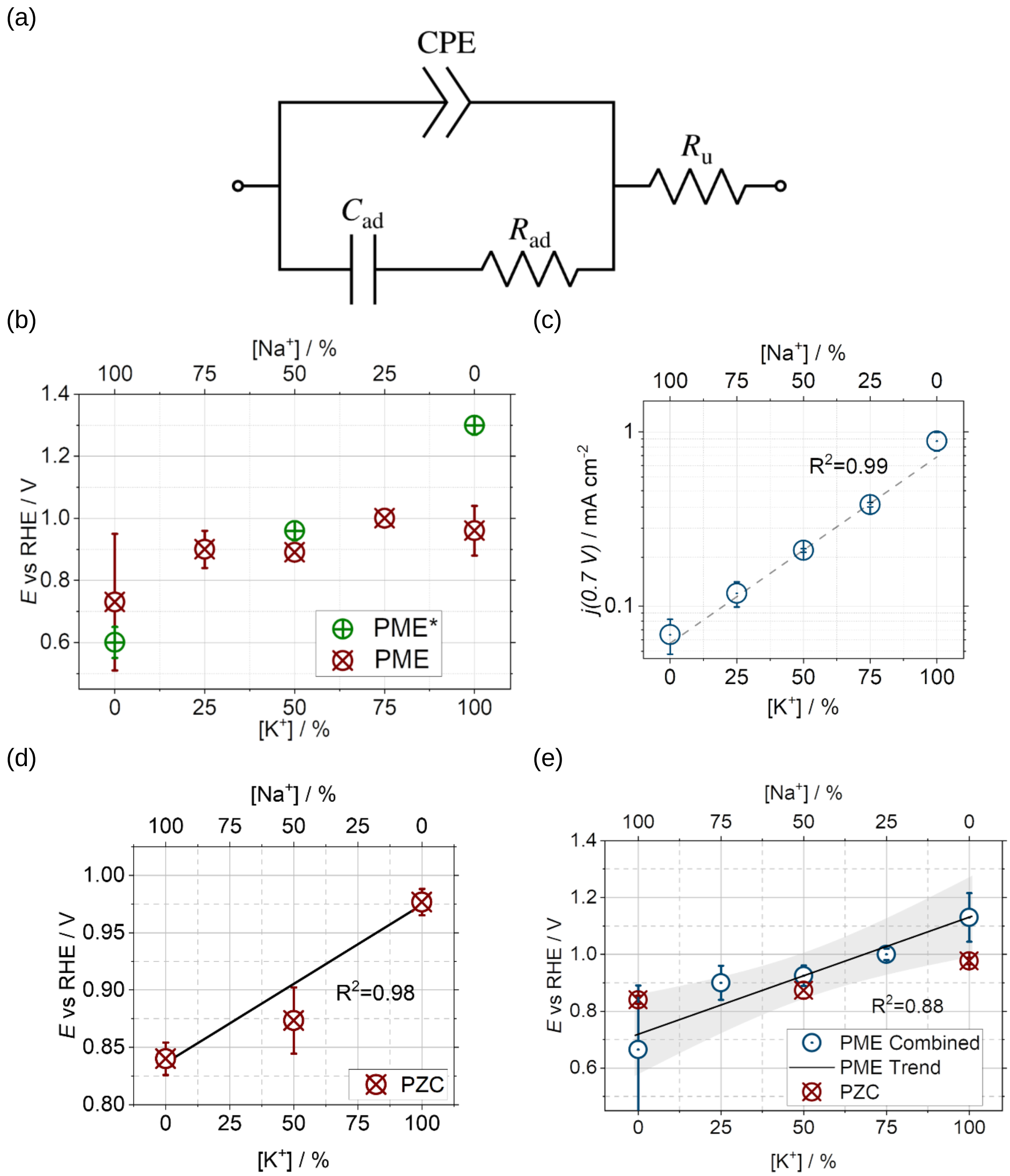

Figure S6. a) The electrical equivalent circuit used to fit the recorded impedance spectra. The CPE modeled the capacitance of the double layer, the resistance of the electrolyte, wires, and other electrical components was represented by the uncompensated resistance R_U. The adsorption processes were modeled by an RC element. b) Representative plot of the PMEs *vs*. RHE obtained in this work and the reference study[5] against the cation (Na^+, K^+) molar ratios. The PME from the reference study is marked with * and indicated with green color plus a sign embossed in a circle. The PME evaluated in this work is portrayed in red as a cross in a circle c) Measured current densities evaluated at 0.7 V_{RHE} obtained for the cation mixtures for the merged results (reference study and this work) d) PZC plotted against the electrolyte composition (cation molar ratios). The larger the relative K^+ concentration of the electrolyte, the higher the PZC. e) Combined PME measurements and the PZC measured by Ding *et al*. [5] and in this work.

References

[1] Spectra Physics, Pulsed Nd and: YAG and Laser User's Manual, Quanta Ray INDI, 2001.
[2] A. V. Himmelreich, Master's thesis, TUM ECS, November **2023**.
[3] X. Ding, PhD thesis, TUM ECS, **2021**.
[4] D. Scieszka, C. Sohr, P. Scheibenbogen, P. Marzak, J. Yun, Y. Liang, J. Fichtner, & A. S. Bandarenka, ACS Appl Mater Interfaces. **2018**, *10*, *25*, 21688-21695.
[5] X. Ding, B. Garlyyev, S. A. Watzele, T. K. Sarpey, & A. S. Bandarenka, *Chemistry - A European J.* **2021**, *27*, *39*, 10016-10020.
[6] D. Sciezska, PhD thesis, TUM ECS, **2019**.
[7] A. Auer, X. Ding, A.S. Bandarenka, & J. Kunze-Liebhäuser, J. Phys. Chem. C Nanomater. Interfaces **2021**, *125*, *9*, 5020-5028.
[8] V. A. Benderskii & G. I. Velichko, J. Eectroanal. Chem. **1982**, *140*, *1*, 1-22.
[9] N. García-Aráez, V. Climent, & J. M. Feliu, Electrochimica Acta, **2009**, *54*, *3*, 966-977.
